# P02.62. DHEA augmentation strategy for treatment of fatigue and depression: a case presentation

**DOI:** 10.1186/1472-6882-12-S1-P118

**Published:** 2012-06-12

**Authors:** R Glick, X Jimenez

**Affiliations:** 1 University of Pittsburgh School of Medicine, Pittsburgh, USA

## Purpose

Dehydroepiandrosterone (DHEA) and its sulfated derivative DHEA-S are endogenous hormones secreted by the adrenal cortex in response to ACTH and stress. Levels of DHEA and DHEA-S are decreased with advancing age and depression. Trials of DHEA for mid-life depression have shown promising yet inconclusive results, due in part to small sample sizes and lack of placebo comparators. An additional concern, with the risk of breast cancer in this age group, is the high doses of DHEA supplementation used. We present a case in which low-dose topical DHEA and other hormonal agents were used as an adjunct to conventional antidepressant therapy to target symptoms of depression and fatigue in a peri-menopausal woman.

## Methods

Baseline levels of DHEA-S were collected. In addition, we assessed fatigue and depression using PROMIS measures and vitality based on the SF-36. DHEA/Pregnenolone/Progesterone topical cream, dosed at 10/20/50 mg/mL per day, was added to the patient’s usual treatment regimen of fluoxetine 20mg daily.

## Results

Baseline labs revealed DHEA-S level of 63 (ref range: 15-170). Baseline questionnaires revealed fatigue at 25/35 (moderate-severe), depression at 23/40 (moderate), and vitality at 8/24 (low). After 12 months of treatment, DHEA-S was slightly increased at 83 and rating scores included fatigue at 11 (mild), depression 10 (mild) and vitality at 19 (high). A decrease of fluoxetine to 10 mg daily was well tolerated and no adverse effects were seen with treatment.

## Conclusion

Given its safety and efficacy profile, low-dose topical DHEA adjunctive treatment for fatigue and depression in mid-life may result in well-tolerated improvements in subjective measures of fatigue, depression, and vitality, and warrants further investigation. Future investigation in a placebo-controlled, RCT is warranted. Such studies should assess safety and tolerability, effect on downstream hormones such as estradiol, optimal dosing, and impact on mood.

